# Amniotic membrane-encapsulated chitosan–lecithin nanoparticles promote the regenerative potential of mesenchymal stromal cells and fibroblasts†

**DOI:** 10.1039/d5na00222b

**Published:** 2025-06-26

**Authors:** Ahmed M. Abou-Shanab, Mostafa Fytory, Shaimaa Shouman, Dina Atta, Asmaa Khairy, Radwa Ayman Salah, Omaima Idris, Nagwa El-Badri

**Affiliations:** a Center of Excellence for Stem Cells and Regenerative Medicine, Zewail City of Science and Technology Giza 12578 Egypt nelbadri@zewailcity.edu.eg; b Biomedical Sciences Program, University of Science and Technology, Zewail City of Science and Technology Giza 12578 Egypt; c Material Science and Nanotechnology Department, Faculty of Postgraduate Studies for Advanced Sciences (PSAS), Beni-Suef University Beni-Suef 62511 Egypt; d Fetal Medicine Unit, Department of Obstetrics and Gynecology, Cairo University Cairo Egypt

## Abstract

The human amniotic membrane (hAM) is a promising therapeutic source in regenerative medicine due to its rich content of growth factors and bioactive molecules. Its clinical application, however, has remained limited by challenges in controlled delivery and compositional complexity. To address these issues, we developed a nanoparticle-based formulation (NF-hAM) to encapsulate hAM proteins and enhance their targeted delivery to mesenchymal stem cells (MSCs) and fibroblasts. NF-hAM was synthesized using soy lecithin and chitosan, forming spherical nanoparticles with well-defined morphology. Physicochemical characterization confirmed successful protein encapsulation and nanoparticle stability. *In vitro* studies demonstrated that NF-hAM was biocompatible and significantly promoted the proliferation of bone marrow-derived MSCs (BMSCs), adipose-derived MSCs (AMSCs), and human skin fibroblasts (HSFs). In HSFs, NF-hAM also induced cytoskeletal remodeling, suggesting enhanced cellular activity and functional responsiveness. Additionally, NF-hAM enhanced the osteogenic and adipogenic differentiation potential of BMSCs. Metabolic analyses further revealed that NF-hAM stimulated glycolytic activity, a key metabolic pathway associated with self-renewal and proliferation. Our findings highlight the potential of NF-hAM as a therapeutic nanomedicine for regenerative medical applications.

## Abstract 2

Development of a chitosan-soy lecithin nanoparticle-based formulation (NF-hAM) for controlled delivery of human amniotic membrane (hAM) proteins to mesenchymal stem cells (MSCs) and fibroblasts, promoting cellular proliferation and differentiation.

## Graphical abstract

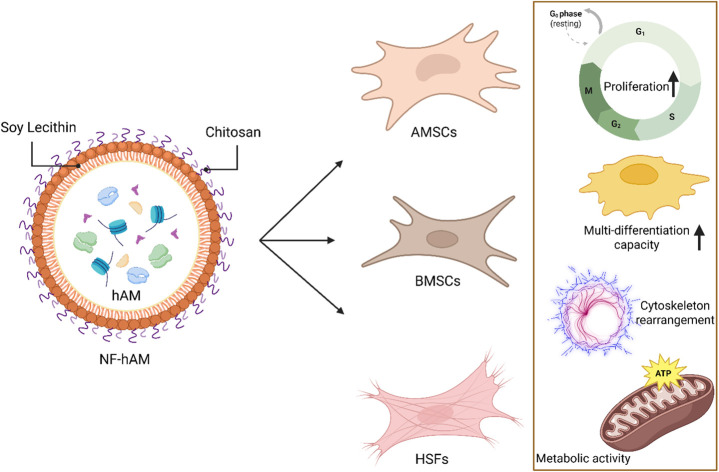

## 1. Introduction

A significant number of diseases affecting human health are awaiting the development of successful cell-based therapies. Recent therapeutic approaches, such as stem cell (SC) therapy, have been proposed to restore tissue function by harnessing the regenerative capacity of the body and enhancing its repair mechanisms.^1^ SCs sustain high regenerative capacities due to their multi-differentiation potential and robust secretion of growth factors.^2–4^ These regenerative capacities were, however, reported to be compromised by multiple factors, including aging and various diseases.^5^ Enhancing the compromised SC characteristics to maximize their therapeutic effect has thus been the subject of extensive research.

Novel usage of natural substrates for enhancing SC regenerative capacities is an attractive approach that has recently been the subject of great interest.^6–8^ The human amniotic membrane (hAM) is a transparent, avascular layer of the placenta that surrounds the fetus during pregnancy. It lacks lymphatic vessels, nerves, and muscles and is composed of an epithelial layer, a basement membrane, and an avascular stromal matrix. Its unique biological composition, including anti-tumorigenic, antimicrobial, and pro-regenerative properties, makes it a valuable source for regenerative medicine.^9^ Moreover, hAM is an accessible and ethically non-controversial by-product of childbirth that is often discarded in hospitals.^10,11^ hAM has gained popularity for its use in the treatment of skin burns and grafts,^12,13^ ophthalmology,^10,11^ and surgeries of the head, neck, genitourinary tract, oral cavity, and stomach.^12,14^ hAM is known to have anti-inflammatory, anti-angiogenic, and anti-microbial effects.^15–17^ hAM has been reported to comprise two types of stem cells, hAM epithelial cells (AECs) and hAM mesenchymal stem cells (AMCs), which were reported to preserve the ability to differentiate into cells from all three germ layers.^18,19^

Multiple forms of the hAM have been used in research and clinical applications.^20^ Decellularized hAM (d-hAM), where the AEC layer of the hAM is denuded, has previously been applied to the culture of BMSCs, promoting their proliferation and differentiation into adipogenic and osteogenic lineages.^21^ We have previously reported that AMSCs cultured on d-hAM exhibited an increased proliferation rate, upregulation of stemness factors, and inhibition of apoptosis, as well as modulation of neo-angiogenic factors and inflammatory marker expression in these cells.^22^ Umbilical cord blood mononuclear cells (MNCs), HSFs, and microvascular endothelial cells cultured on hAM-coated plates have also shown higher survival and proliferation rates without inducing any cytotoxic effects.^8,23^ Additionally, hAM extracts, derived from d-hAM, have shown immense potential as a valuable resource in regenerative medicine owing to their potent bioactive content,^15,24,25^ making them a subject of considerable research interest.^26–29^ However, hAM clinical applications in regenerative medicine have some limitations due to its complex composition and constraints on its controlled delivery. The risk of infections and the complex process of inserting the hAM graft during treatment, coupled with a reduction in the number of bioactive factors following surgery, are also major concerns.^30^ This may compromise the use of hAM for optimal tissue regeneration, which mandates precise, timed, and localized delivery, essential to induce the desired cellular response.^31^

Nanoparticles (NP) of diverse characteristics are currently employed to target stem cells with molecules that are capable of enhancing their regenerative capacities. These NPs have been approved for clinical use.^31,32^ NPs designed for regenerative medicine applications possess specific properties, such as being biocompatible with low cytotoxicity and not triggering the immune response.^33^ Moreover, these NPs should be biodegradable, chemically stable under physiological conditions, and guarantee efficacy at therapeutic doses.^34^ For effective SC-targeted-NPs, SCs' properties must also be preserved, including their proliferative, differentiation, immunomodulatory, migratory, and homing capabilities.^31^ Many types of NPs have been reported for targeting SCs with small molecules, to protect small molecules from premature degradation and ensure their delivery. NP materials are used for delivering different small molecules, including polymers, lipids, and metals.^35^ Polymeric NPs have shown great potential as drug carriers for the treatment of several diseases, due to their small size, stability, safety, and controlled release, which improves the bioavailability of the loaded molecules, absorption, protection from degradation, and targeted delivery.^36,37^ Notable examples of natural polymers include chitosan, known for its unique biocompatible, biodegradable, and mucoadhesive characteristics,^38^ and lecithin, a cost-effective food-grade surfactant.^39^ Nevertheless, challenges have been reported, including inadequate stability, poor electrical and mechanical properties of chitosan, and the formation of lipid hydroperoxides by lecithin. This results in impairment of their bioactivity and potential cell toxicity.^40,41^ Therefore, many attempts to develop successful SC-targeted-NPs have focused not only on their non-toxic effects on cells, but also on their capability of preserving SC regenerative capacities, to improve their direct contribution to the regeneration process.^42,43^

In this work, we aim to develop biocompatible hAM NP-based formulations (NF-hAM) to encapsulate hAM proteins ensuring their delivery to MSCs and HSFs to enhance their functionality and regenerative capacities.

## 2. Material and methods

### 2.1 Collection of the hAM

hAM was collected after elective cesarean sections from healthy subjects screened negative for blood-borne infections at Sheikh Zayed Specialized Hospital. Our protocol was reviewed and approved by the Institutional Review Board (IRB), and ethical approval (ZU-IRB #11403) and informed consent forms were obtained before the collection of samples. hAM samples were then placed in a sterile saline solution and immediately transported to the laboratory. The amnion was manually and gently stripped from the chorion membrane. The separated hAM was then placed on ice and thoroughly washed with phosphate-buffered saline (PBS) supplemented with 2% antibiotic/antimycotic solution to remove blood and debris. Following the washing process, each hAM sample was minced, and 5 grams of tissue from each sample were collected. Following previously optimized protocols (Mamedo *et al.*^44^ and our protocol in ref. 45), hAM tissues were homogenized and sonicated on ice to prevent protein denaturation. This was followed by centrifugation at 17 000 × *g* for 20 minutes at 3 °C. The supernatant was collected, and protein concentrations were measured using the Bradford assay. The extracted protein solutions were then stored at −80 °C until lyophilization.

### 2.2 Nano-formulation preparation

Soy lecithin (Epikuron™ 200, Cargill, Germany) and low molecular weight chitosan (Sigma Aldrich, USA) were used to prepare the hAM NPs, using electrostatic interactions *via* ionic gelation. This approach involved the direct injection of an ethanolic lecithin solution into an aqueous chitosan solution, based on the method described previously, with some modifications.^46^ In brief, a chitosan solution was made in 1% (w/v) acetic acid and stirred overnight at 25 °C until it fully dissolved. Lecithin–chitosan NPs with hAM extract were produced by dripping 2 mL of a 50 mg per mL lecithin ethanolic solution (containing 5 mg of hAM) into 10 mL of a 2 mg per mL chitosan aqueous solution. This was done at a flow rate of 1 mL min^−1^ using a 0.38 mm syringe, under sonication, followed by continuous magnetic stirring at 1000 rpm. After the ethanol was completely evaporated through stirring, the solution was centrifuged at 10 000 rpm for 15 minutes, washed three times with deionized water, and the final samples were freeze-dried. The free nanoformula was synthesized with the same procedure without adding the hAM extract.

### 2.3 Characterization of NPs

Transmission electron microscopy (TEM) analysis was performed by suspending the NPs in an aqueous medium, which was then deposited onto a grid for imaging using a JOEL-JEM2100 microscope (Akishima, Japan).

Fourier-transform infrared (FT-IR) spectra were acquired with a Shimadzu 470 spectrophotometer, covering a range of 4000 to 400 cm^−1^.

Thermogravimetric analysis (TGA) of the NPs was conducted using a PerkinElmer STA6000 analyzer; the samples were heated from room temperature to 600 °C at 10 °C min^−1^ under a nitrogen atmosphere.

Additionally, dynamic light scattering (DLS) measurements were performed to determine the particle size and *ζ*-potential of the nanocomposites using a Zetasizer Nano ZS (Malvern, UK).

### 2.4 Determination of hAM encapsulation efficiency (EE%)

The encapsulation efficiency (EE%) of hAM-loaded NPs was assessed using an indirect method. Initially, 4 mg of hAM was dissolved in 2 mL of PBS, and its concentration was determined using ultraviolet-visible (UV-vis) spectrophotometry with a calibration curve at 278 nm. Subsequently, the concentration of hAM in the resulting supernatant was measured at the maximum wavelength (*λ*_max_) of 278 nm using UV-vis spectroscopy (Evolution UV 600, Thermo Scientific). Each measurement was performed in triplicate. The EE% of hAM was calculated using the following equation:
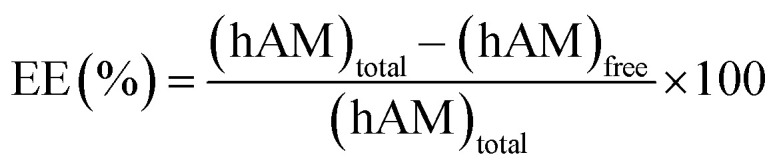
where (hAM)_total_ is the total weight of hAM extract that was initially added to the NF aqueous suspension, while (hAM)_free_ is the amount of free (non-entrapped) hAM in the supernatant.

### 2.5 Cell culture

The BMSC cell line (ATCC, Manassas, VA, USA), adipose-mesenchymal stem cells (AMSCs) (a gift from the Urology and Nephrology Center, Mansoura University, Egypt),^47–49^ and human skin fibroblasts (HSFs) (ATCC, Manassas, VA, USA) were maintained in a complete culture medium (CCM) consisting of DMEM supplemented with 10% FBS and 1% streptomycin/penicillin at 37 °C in a humidified 5% CO_2_ incubator.

Cells were cultivated under different experimental conditions: (i) Ctrl: CCM, (ii) NF-hAM: cells treated with NF-hAM prepared by sonication at a concentration of 5 μg mL^−1^ for 2 days, (iii) NF-free hAM: cells treated with the nano-formula without hAM at a concentration of 5 μg mL^−1^ for 2 days, and (iv) hAM: cells treated with hAM at a concentration of 5 μg mL^−1^ for 2 days.

### 2.6 MTT assay

BMSCs, HSFs, and AMSCs were seeded at a concentration of 20 000 cells per well in 12-well plates in Ctrl, NF-hAM, NF-free hAM, and hAM at a concentration of 5 μg mL^−1^ for 48 hours. To analyze dose dependence, the proliferation of HSFs and BMSCs was tested through 24, 48, or/and 72 hours. BMSCs were assessed at concentrations of 5 and 50 μg mL^−1^, while HSFs were assessed at concentrations of 5, 10, and 50 μg mL^−1^ of NF-hAM and NF-free hAM. Cells were imaged and presented corresponding to the MTT analysis.

Following the treatment, 100 μL of 5 mg mL^−1^ MTT reagent 3-(4,5-dimethylthiazol-2-yl)-2,5-diphenyltetrazolium bromide (Life Technologies, USA) was added to each well and incubated for 3 hours in a humidified chamber at 37 °C and 5% CO_2_. 500 μL of dimethyl sulfoxide (DMSO) was then added and shaken in the dark for 20 min to dissolve the formed formazan salts. The plates were read at 570 nm optical density (OD) using a FLUOstar Omega-microplate reader (BMG Labtech, Cary, NC, USA).

### 2.7 Flow cytometry analysis

BMSCs were collected and washed with PBS, then fixed in 3 mL 75% ethanol and then stored at −20 °C for 3 days.^49^ The cells were then pelleted, washed with PBS twice, and resuspended again in 100 μL ribonuclease A enzyme (100 μg mL^−1^) and incubated for 15 min at room temperature. 200 μL propidium iodide was added and incubated for 60 min at 4 °C in the dark. Lastly, the cells were resuspended in 0.5 mL flow cytometry (FACS) buffer and analyzed for cell cycle progression.

The resistance of BMSCs to cellular apoptosis following NF-hAM and NF-free hAM treatments was analyzed by flow cytometry using an Annexin V FITC and PI apoptosis kit (Miltenyi Biotec Inc., Auburn, CA, USA) as per the manufacturer's protocol. FACSCalibur (Becton Dickinson) was used according to the standardized protocol of CellQuest Pro Software (Becton Dickinson). Data were analyzed using FlowJo v. 10.2 software (Treestar, Ashland, OR, USA).

### 2.8 Analysis of biochemical reprogramming

The amount of glucose and lactate in BMSC and HSF conditioned medium from Ctrl, NF-hAM, and NF-free hAM groups was measured. Glucose was measured using a commercial glucose enzymatic colorimetric assay kit (Biodiagnostic, Cairo, Egypt), while lactate was quantified using a commercial Lactate Plus-Liquizyme enzymatic colorimetric assay kit (catalog number 274 001, Spectrum Diagnostics, Cairo, Egypt). Hydrogen peroxide quantification kit (Biodiagnostic, Egypt) was used to quantify the produced H_2_O_2_ in the conditioned medium of HSFs. The protocol was carried out according to ref. 50 and the manufacturer's instructions at optical densities (ODs) of 510 nm and 546 nm, respectively, using a FLUOstar Omega-microplate reader (BMG Labtech, Cary, NC, USA).

### 2.9 Assessment of BMSC adipogenic and osteogenic differentiation capacities

#### 2.9.1 Adipogenic induction of BMSCs

BMSCs from Ctrl, NF-hAM, and NF-free hAM experimental groups were transferred into the differentiation medium consisting of DMEM (1 g per L glucose), 1% penicillin/streptomycin, 10% FBS, 5 μg per mL insulin, 50 μM indomethacin, 1 μM dexamethasone, and 0.5 μM 3-isobutylmethylxanthine (IBMX). The medium was changed every 2 to 3 days for 10 days. A control group was added to ensure the differentiation medium efficacy, including BMSCs cultured in CCM. After 10 days of adipogenic induction, BMSCs from the four groups were washed thrice with PBS, fixed with 10% formalin, and stained with 1.5 mg per mL Oil Red O (Sigma-Aldrich, USA) in 60% isopropanol for 15 minutes at room temperature. The samples were examined using an inverted microscope (Leica DMi8 Microsystems, Wetzlar, Germany).^51,52^

#### 2.9.2 Osteogenic induction of BMSCs

BMSCs from Ctrl, NF-hAM, and NF-free hAM experimental conditions were transferred into the differentiation medium consisting of DMEM (1 g per L glucose) with 10% FBS, 1% penicillin/streptomycin, 50 μg per mL ascorbic acid (ASA), 10 mM *β*-glycerophosphate, and 100 nM dexamethasone. The medium was changed every 2 to 3 days for 12 days. A control group was added to ensure the differentiation medium efficacy, including BMSCs cultured in CCM. For Alizarin Red-S staining, BMSCs from the four groups were fixed with 70% ethanol for 2 hours. After washing with PBS, the cells were stained for 1 hour with 13 mg per mL Alizarin Red-S (pH 4.1; Sigma-Aldrich) and thereafter washed with distilled H_2_O 5 times. The extracellular calcium mineralization was observed by using an inverted microscope (Leica DMi8 Microsystems, Wetzlar, Germany).^51,53^

### 2.10 Immunocytochemistry

To analyze cytoskeletal arrangement, HSFs were seeded onto glass slides and treated as previously described.^33,50^ Subsequently, the cells were fixed, permeabilized with 1% Triton-X 100, and blocked with 1% BSA to prevent non-specific antibody binding. Immunofluorescence staining was then performed using antibodies against β-actin (Cell Signaling, USA) and α-smooth muscle actin (α-SMA) (Invitrogen, USA). Following incubation with appropriate secondary antibodies, cell nuclei were counterstained with Hoechst 33 342 (Molecular Probes, USA). Finally, the stained cells were visualized and imaged using a Leica DMi8 inverted fluorescent microscope.

### 2.11 RT-qPCR

Total RNA was extracted from BMSCs from NF-hAM, NF-free hAM, and Ctrl groups using TRIzol (Sigma-Aldrich, St. Louis, MO, USA) according to the manufacturer's protocol. The purity and concentration of the extracted RNA were assessed at 260/280 nm using a NanoDropTM 2000/2000c spectrophotometer (Thermo Scientific, MA, USA). cDNA synthesis was performed using a RevertAid First Strand cDNA Synthesis Kit (Thermo Fisher, USA) as per the manufacturer's instructions. Quantitative PCR analysis was carried out in triplicate for each cDNA sample using a HERA PLUS SYBR® Green kit (Willowfort, UK). Reactions were run by using a real-time PCR system (CFX96 Touch™ Real-Time PCR Detection System). Relative mRNA quantities were analyzed by using the delta–delta CT (−ΔΔ*C*_t_) method. β-Actin was used for normalization as a reference gene. The sequences of all primer sets are identified in ESI 1, Table S1.†

### 2.12 Statistical analysis

Data are presented as the mean ± SD using GraphPad Prism version 10.2.3. An unpaired two-tailed Student's *t*-test was used to analyze the statistical significance between 2 groups. For comparisons between more than 2 groups, a one-way ANOVA test was used. *P*-values: *p < 0.05, **p < 0.01, ***p < 0.001 and ****p < 0.0001 were considered statistically significant.

## 3. Results

### 3.1 Characterization of the nano-formulations

**Fig. 1:**
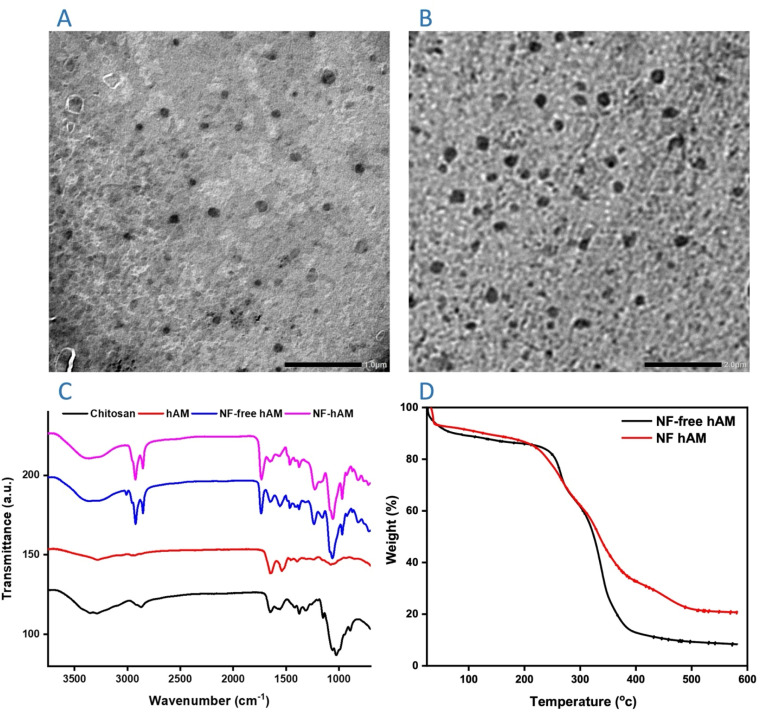
Characterization of the NPs. TEM images (A) NF-free hAM and (B) NF-hAM of the morphology and dispersion of the NPs. FT-IR spectra (C) of raw materials used in NPs preparations and prepared NPs: chitosan (black line), hAM (red line), NF-free hAM (blue line), and NF-hAM (pink line) (D) TGA curve for NF-free hAM and NF-hAM.

TEM was employed to investigate the shape, size distribution, and structural integrity of the developed chitosan–lecithin lipoplex nanoparticles. The TEM images (Fig. 1A and B) demonstrated that free and loaded nanoparticles generally possessed a spherical morphology with a consistent size distribution. A well-defined core–shell structure was identified, demonstrating the successful complexation of chitosan and lecithin. The average particle size, as determined by TEM analysis, was in the range of 90–140 nm, as detected using ImageJ software, which is consistent with dynamic light scattering (DLS) measurements. The absence of large aggregates in the TEM images further confirmed the colloidal stability of the lipoplex nanoparticles. Moreover, the smooth and intact surface morphology indicated successful electrostatic interactions between chitosan and lecithin, promoting nanoparticle formation.

The FT-IR spectra of the NPs, both with and without hAM (Fig. 1C), displayed a prominent absorption band between 3200 and 3500 cm^−1^, corresponding to NH stretching. Bands observed at 1652, 1590, and 1380 cm^−1^ were indicative of NH bending within glucosamine units. An absorption peak at 1150 cm^−1^, associated with the asymmetric stretching of the C–O–C bond, is characteristic of a saccharide structure. These peaks are consistent with the structure of chitosan, suggesting that chitosan is present on the surface of the nanoparticles. Two additional absorption peaks were noted for hAM at 3285 and 1654 cm^−1^, which correspond to O–H and C=C stretching, respectively, indicating the presence of alcohols, phenols, and conjugated alkenes in the lyophilized hAM.

TGA is crucial for assessing thermal stability and compound interactions, as illustrated in Fig. 1D. The thermogram of hAM displayed a minor weight loss around 100–127 °C, corresponding to the dehydration phase relative to the total mass.^54^ NF-free hAM experienced an 80% mass reduction between 292 and 400 °C. Incorporating hAM within the nanostructure (NF-hAM) improved its thermal stability.

The NPs demonstrated a size of 289.9 nm with a polydispersity index (PDI) of 0.22 for NF-free hAM, while NF-hAM had a size of 189.7 nm with a PDI of 0.23, indicating an efficient synthesis process with minimal steps. All samples exhibited PDI values below 0.24 (*p* > 0.05), reflecting a narrow size distribution.

The zeta potential reflects the surface charge of particles in suspension and plays a key role in determining the stability of the dispersion. Particles with zeta potentials greater than +30 mV or less than −30 mV are generally considered stable and less prone to aggregation or destabilizing interactions such as van der Waals forces, Brownian motion, or particle–particle interactions.^10^ The NF-free hAM and NF-hAM samples each exhibited cationic surface characteristics, as indicated by their respective mean zeta potential values of +31.8 ± 3.6 mV and +17.1 ± 5.4 mV.

### 3.2 NF-hAM safety and effectiveness in promoting the proliferation of BMSCs, HSFs, and AMSCs

**Fig. 2:**
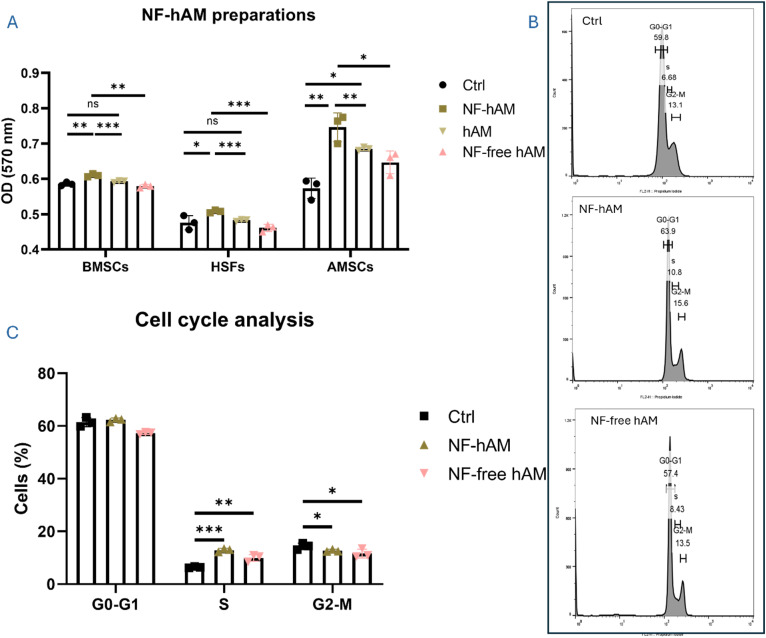
NF-hAM safety and effect on BMSCs, HSFs, and AMSCs proliferation. (A) MTT analysis of BMSCs, AMSCs, and HSFs under different experimental conditions (*n* = 3). (B) Flow cytometry cell cycle analysis of BMSCs. (C) Bar graphs indicate BMSC population percentages in different cell cycle phases (*n* = 3).

Our results demonstrate that NF-hAM significantly stimulated the proliferation of all three cell types. The presence of hAM within the nanoparticles was confirmed by comparing the effects of NF-free hAM and NF-hAM on cell proliferation. NF-hAM significantly enhanced proliferation in BMSCs, HSFs, and AMSCs compared to NF-free hAM (Fig. 2A).

MTT assays revealed that treatment with 5 μg mL^−1^ NF-hAM significantly enhanced the proliferative capacity of BMSCs, HSFs, and AMSCs (Fig. 2A). Cell cycle analysis of BMSCs in the NF-hAM and NF-free hAM groups demonstrated a marked increase in the S phase population compared to the Ctrl group, further supporting their enhanced proliferation (Fig. 2B and C).

### 3.3 Protective effect of NF-hAM against BMSC apoptosis

**Fig. 3:**
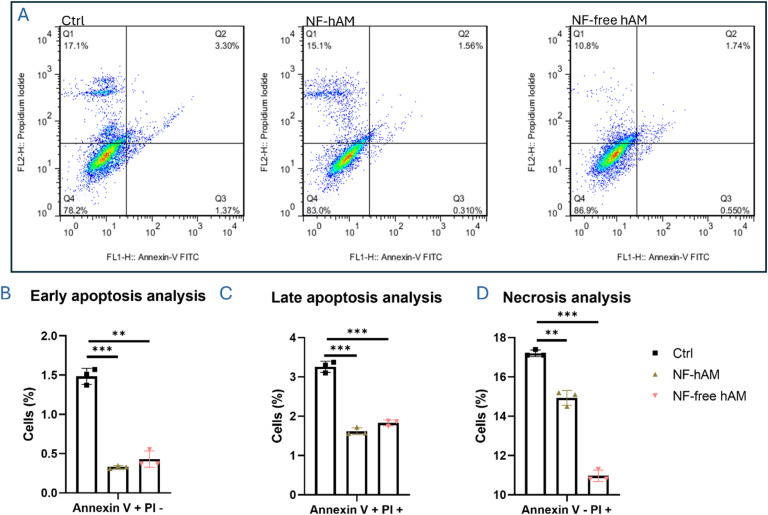
Analysis of BMSCs' potential to resist apoptosis induction. (A) Annexin V/PI staining analysis by flow cytometry. (B) Bar graph depicting BMSC in early apoptosis (*n* = 3). (C) Analysis of BMSCs in the late apoptosis phase (*n* = 3). (D) Analysis of BMSC percentage in the necrosis phase (*n* = 3).

To evaluate the safety of NF-hAM on BMSCs, we assessed their resistance to apoptosis induction. NF-hAM treatment resulted in a significant decrease in the BMSC population in both early (Annexin V+/PI−) and late (Annexin V+/PI+) apoptosis phases compared to the control group (Fig. 3A–C). Additionally, analysis of cellular necrosis revealed a significant reduction in the BMSC number in the Annexin V−/PI + quartile of the NF-hAM and NF-free hAM groups compared to the control (Fig. 3A and D).

We also investigated the potential cytoprotective effect of NF-hAM from induced oxidative stress. HSF cells were exposed to H_2_O_2_ treatment at a final concentration of 300 μM for 12 hours, followed by 48 hours of NF-hAM treatment. Our MTT analysis demonstrated a significantly higher proliferation rate in the NF-hAM group as compared to H_2_O_2_-treated cells (ESI 1, Fig. S1†).

### 3.4 NF-hAM promotes the proliferation of BMSCs and HSFs in a dose-dependent manner

**Fig. 4:**
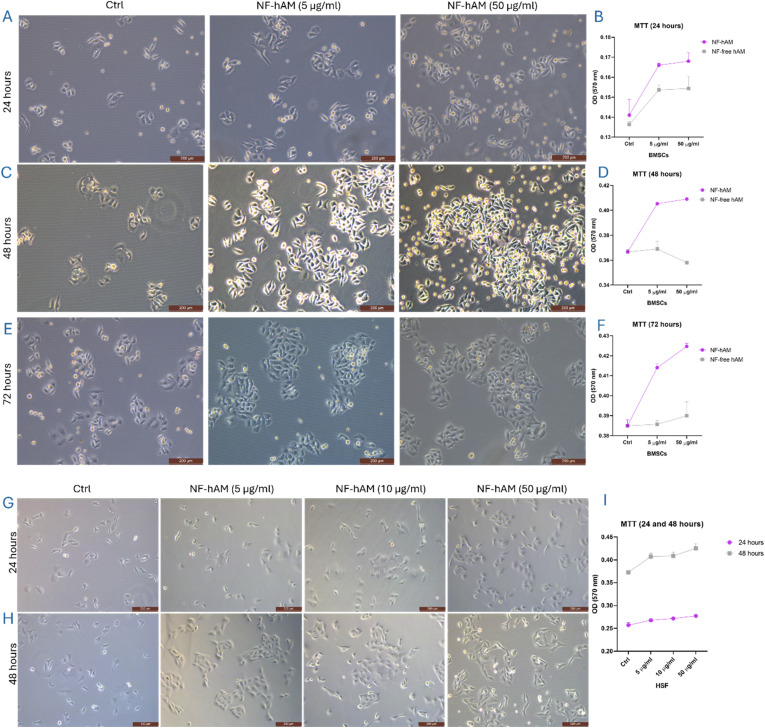
Effect of NF-hAM on BMSCs and HSFs proliferative capacity. (A) BMSC treated NF-hAM for 24 hours (scale bar: 200 μm). (B) MTT assay for BMSCs treated with NF-hAM for 24 hours (*n* = 3). (C) BMSC treated NF-hAM for 48 hours (scale bar: 200 μm). (D) MTT assay for BMSCs treated with NF-hAM for 48 hours (*n* = 3). (E) BMSC-treated NF-hAM for 48 hours (scale bar: 200 μm). (F) MTT assay for BMSCs treated with NF-hAM for 72 hours (*n* = 3). (G) HSFs-treated NF-hAM for 24 hours (scale bar: 200 μm). (H) HSFs-treated NF-hAM for 48 hours (scale bar: 200 μm). (I) MTT assay for HSFs-treated NF-hAM for 24 and 48 hours (*n* = 3).

To evaluate the efficacy of NF-hAM on BMSC proliferation, they were treated with varying concentrations (5 and 50 μg mL^−1^) and treatment durations (24, 48, and 72 hours). NF-hAM treatment for 24 hours significantly stimulated BMSC proliferation at both 5 and 50 μg mL^−1^, with a more pronounced effect observed at the higher concentration (Fig. 4A and B). This enhanced proliferation was not observed in the NF-free hAM group, suggesting the effective delivery of hAM proteins to BMSCs (Fig. 4A and B). Similar results were obtained with NF-hAM treatment for 48 and 72 hours at both 5 and 50 μg mL^−1^ (Fig. 4C–F).

Moreover, we examined the effect of different concentrations of NF-hAM (5, 10, and 50 μg mL^−1^) on HSFs for 24 hours (Fig. 4G) and 48 hours (Fig. 4H). NF-hAM treatment for 24 hours significantly enhanced HSF proliferation at 5, 10, and 50 μg mL^−1^, with a more pronounced effect observed at the higher concentration (Fig. 4A and B). The MTT proliferation assay showed a significant increase in HSF proliferation by increasing the NF-hAM dosage or duration of the treatments (Fig. 4I).

### 3.5 NF-hAM promotes the multi-lineage differentiation capabilities of BMSCs

**Fig. 5:**
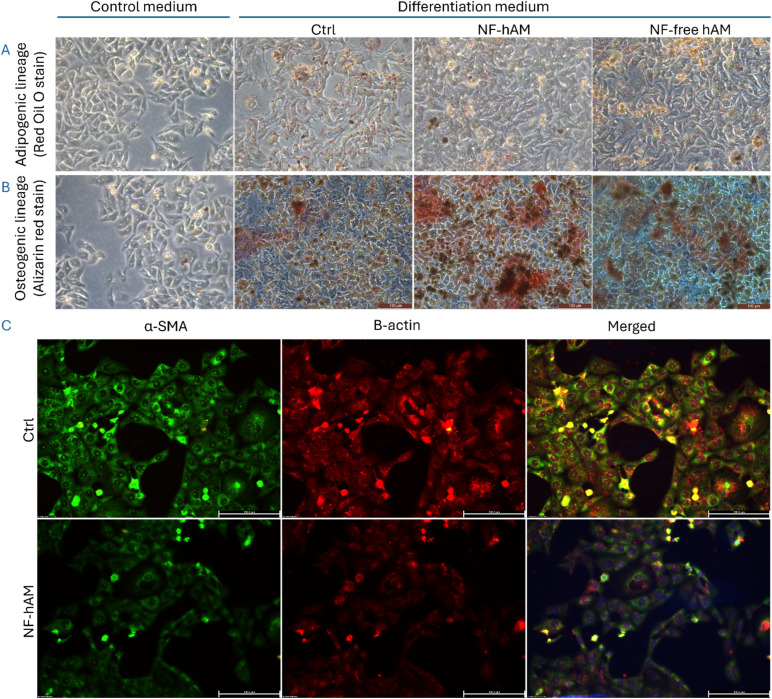
Enhancement of BMSC adipogenic and osteogenic differentiation capacity and effect of NF-hAM on HSFs morphology. (A) BMSCs stained with Red Oil O stain upon cultivation in adipogenic induction medium (scale bar: 100 μm). (B) BMSCs stained with Alizarin Red Stain upon cultivation in osteogenic induction medium (scale bar: 100 μm). (C) Immunofluorescence staining of cytoskeletal α-SMA and β-actin (scale bar: 155.5 μm).

To investigate the impact of NF-hAM on BMSC differentiation, we assessed both osteogenic and adipogenic pathways (Fig. 5A and B). We treated the cells in experimental groups: CCM, NF-hAM, and NF-free hAM. Then, we cultivated BMSCs from the three different groups in the differentiation medium and included another control CCM group under regular CCM conditions for 10–12 days. Oil Red O staining, used to visualize lipid droplets, revealed no significant difference in adipogenic differentiation between the NF-hAM, NF-free hAM, and control groups after 10 days of induction (Fig. 5A).

BMSCs treated with NF-hAM demonstrated significantly enhanced osteogenic differentiation, as evidenced by Alizarin Red staining of calcium deposits, compared to the Ctrl and NF-free hAM groups after 12 days (Fig. 5B).

### 3.6 NF-hAM effect on HSF cytoskeleton regulation

Immunofluorescence staining of β-actin, a primary component of the cells' internal structure, revealed significant changes in the arrangement of the filamentous network (Fig. 5C). Similarly, α-SMA expression, a marker for contractile stress fibers, was downregulated in NF-hAM-treated cells compared to the Ctrl group (Fig. 5C).

### 3.7 NF-hAM modulates the bioenergetics of MSCs and HSFs

**Fig. 6:**
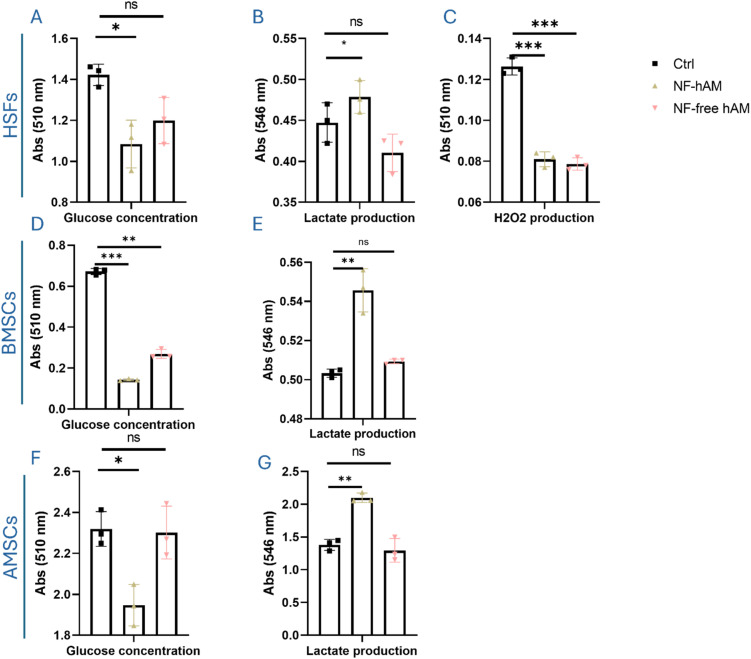
NF-hAM treatment elevated glycolytic metabolism in BMSCs and HSFs. (A) BMSCs conditioned medium glucose quantity. (B) Lactate production in the conditioned medium of BMSCs. (C) H_2_O_2_ production in HSF cells. (D) HSFs conditioned medium glucose quantity. (E) Lactate production in the conditioned medium of HSFs. (F) Conditioned medium glucose quantity in AMSCs. (G) AMSCs' lactate production in the conditioned medium.

Conditioned medium glucose was significantly lower in BMSCs (Fig. 6A), HSFs (Fig. 6D), and AMSCs (Fig. 6F) treated with NF-hAM than their counterparts in the Ctrl group. Lactate production was shown to be significantly upregulated in the NF-hAM-treated cells as compared to the Ctrl group in BMSCs (Fig. 6B), HSFs (Fig. 6E), and AMSCs (Fig. 6G). H_2_O_2_ production was shown to be decreased in HSFs treated with NF-hAM compared to their counterparts in the Ctrl group (Fig. 6C).

### 3.8 NF-hAM modulates BMSCs' proliferation, migration, and angiogenesis molecular marker expression

**Fig. 7:**
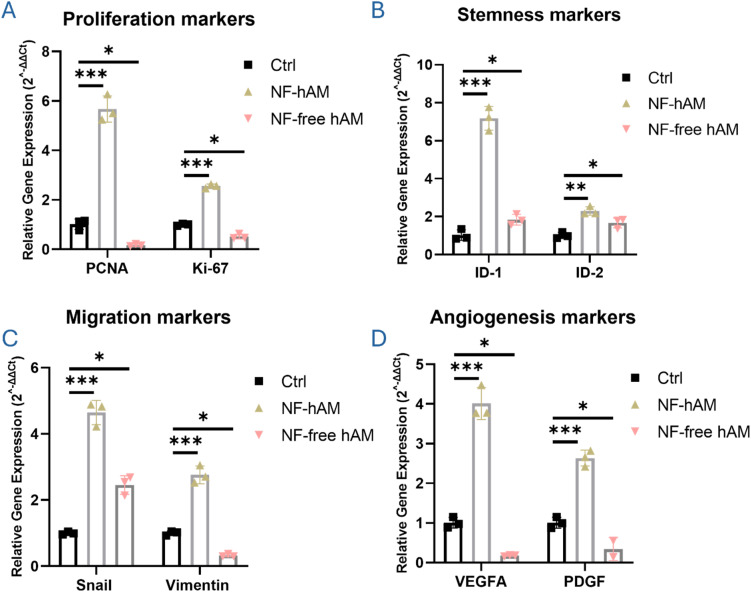
Upregulation of proliferation, angiogenesis, and migration markers genotypic expression in BMSCs upon NF-hAM treatment. (A) PCNA and Ki67 proliferation markers expression. (B) ID-1 and ID-2 stemness and proliferation markers expression in BMSCs. (C) Snail and vimentin mesenchymal markers expression. (D) VEGFA and PDGF angiogenic markers expression.

To evaluate the functional enhancement of BMSCs by NF-hAM, we performed RT-qPCR analysis targeting key molecular markers of proliferation, stemness, migration, and angiogenesis. Proliferation markers PCNA (proliferating cell nuclear antigen) and Ki-67, a nuclear protein expressed during all active phases of the cell cycle, were significantly upregulated in BMSCs treated with NF-hAM compared to the Ctrl group, indicating enhanced proliferative activity (Fig. 7A).

To assess stemness-associated activity, we examined the expression of inhibitor of differentiation (ID)-1 and ID-2. Both ID1 and ID2 were significantly upregulated in the NF-hAM-treated group, suggesting maintenance of a more naïve and proliferative stem cell phenotype (Fig. 7B).

Considering the importance of cell migration and the mesenchymal phenotype in regenerative medicine and wound healing, we evaluated the expression of Snail, a transcriptional repressor promoting epithelial-to-mesenchymal transition (EMT), and vimentin, a structural intermediate filament protein and key marker of mesenchymal identity. NF-hAM treatment significantly enhanced the expression of both markers, indicating an increased migratory and mesenchymal potential of BMSCs (Fig. 7C).

Lastly, to assess angiogenic capacity, we analyzed the expression of VEGFA (vascular endothelial growth factor A), a potent inducer of endothelial cell proliferation and new vessel formation, and PDGF (platelet-derived growth factor), a mitogen critical for angiogenesis and vascular stability. Both genes were significantly upregulated upon NF-hAM treatment compared to the Ctrl group, suggesting enhanced angiogenic signaling potential (Fig. 7D).

## 4. Discussion

Nanomedical applications are revolutionizing healthcare by offering innovative solutions to a wide range of health challenges.^55^ One of the most promising areas of nanomedicine is targeted drug delivery, which involves using NPs to deliver drugs or substances *via* binding to specific molecular targets on the surface of diseased cells or tissues.^55,56^ This targeted approach can significantly improve the efficacy of treatments while minimizing off-target effects.^56^ This targeted delivery can enhance drug bioavailability, reduce systemic toxicity, and improve patient outcomes.^57^ The amniotic membrane is a valuable resource in regenerative medicine, offering a biocompatible scaffold, stem cells, and growth factors.^29,58,59^ We have previously reported the beneficial effects of incorporating the d-hAM into 3D cultures of MSCs, highlighting its potential to promote their regenerative capabilities, thereby influencing their therapeutic potential.^60^ We found that elevated glycolysis and suppressed oxidative metabolism in AMSCs and BMSCs are significantly affected at the molecular and biochemical levels upon d-hAM incorporation into the 3D culture.^60^ In this paper, we aimed to develop hAM protein extract NPs (NF-hAM) to enhance hAM protein delivery into MSCs and HSFs, promoting their functionality.

Soy lecithin and chitosan were used in forming NF-hAM, which was characterized using TEM. According to Souza *et al.*, the formation of lecithin-based and chitosan nanoparticles is driven by the electrostatic interactions between the positive charges of chitosan and the negative charges of lecithin, leading to self-assembly.^61^ Consequently, lecithin forms the core of the spherical nanoparticles, while the chitosan molecules create an outer layer that encapsulates and protects the inner structure.^46^ Then, FT-IR characterization of NF-hAM showed various peaks of the chitosan^62^ and hAM, indicating the presence of alcohols, phenols, and conjugated alkenes in the lyophilized hAM.^63^ The absence of distinct hAM peaks in the spectra of the NPs suggests that hAM is encapsulated within the NPs and that its interactions with them are likely to involve hydrophobic interactions or hydrogen bonding. Additionally, TGA indicated that incorporating hAM within NF-hAM improved its thermal stability (Fig. 3). A PDI value of 0.4 or higher indicates a lack of homogeneity in particle size distribution; therefore, a lower PDI is closely associated with both size and stability.^64^ Achieving small and uniform nanoparticles is critical for preparing stable colloidal dispersions,^65^ as larger particles can promote the formation of Ostwald ripening, leading to coalescence and destabilization of nanosystems.^66^ The zeta potential values suggest that a stable nanoparticle suspension was achieved. The observed positive surface charge indicates the presence of chitosan on the particle surfaces, as nanoparticles coated with chitosan typically exhibit zeta potentials in the range of +30 to +50 mV due to the protonation of amino groups (NH_3_^+^) at acidic pH.^67^ The reduced zeta potential of NF-hAM may result from interactions between hAM extract and the chitosan polymer. A similar result (+31.17 ± 4 mV) was obtained by a study that also used chitosan in the elaboration of lecithin-clotrimazole nanoparticles.^68^

The encapsulation efficiency of the hAM extract was approximately 80%. This efficiency largely depends on the solubility of the molecule, the composition of the polymer or matrix, and the preparation method used. A substantial amount of hAM was successfully encapsulated within the lipid cores of the NPs, while only a minor portion remained soluble in the aqueous dispersion phase, resulting in high encapsulation efficiency. A common technique for NP preparation involves injecting an alcoholic lecithin solution into an aqueous chitosan solution.^62^ Lecithin acts as a surfactant, enhancing the solubility of hAM, which, due to its hydrophobic nature, is incorporated into the negatively charged core of the NPs through strong affinity. The addition of polymer coatings such as positively charged chitosan induces electrostatic attraction, altering the surface charge distribution and thickness of the NPs. This process significantly influences their properties, including solubility, colloidal stability, absorption, and bioavailability.^64^

NPs were synthesized using the ionic gelation method, followed by a comparative analysis of their cytotoxicity and cellular responses in BMSCs, AMSCs, and HSFs. Then, we functionally analyzed the delivery of encapsulated hAM inside the chitosan nanoparticle by studying its effects on the proliferation of AMSCs, BMSCs, and HSFs as compared to NF-free hAM NPs (Fig. 2). As observed, NF-hAM formulation was more effective in delivering hAM proteins to cells compared to the hAM extract alone, as compared to the Ctrl group. Therefore, to focus on evaluating the impact of NP-mediated delivery, we limited the follow-up assays to NF-hAM, NF-free hAM, and Ctrl groups. The increased proliferation of these cells following NF-hAM treatment indicates the nanoparticles' efficacy in delivering hAM proteins. hAM has previously been reported to promote cellular proliferative capacity.^59,60,69,70^ The proliferation of BMSCs upon treatment with NF-hAM was shown to be dose-dependent in response to different concentrations (Fig. 4). In cell therapy and regenerative medicine, incremental improvements in cell proliferation may translate into enhanced therapeutic outcomes.^71,72^ Moreover, NF-hAM was found to promote HSF resistance to apoptosis induction, which further confirms its safety. This is in accord with our previous report in which the hAM was used as a biological scaffold for the culture of BMSCs and AMSCs.^60^

BMSCs are a type of adult stem cell with the ability to self-renew and differentiate into various cell types, including bone, cartilage, fat, and muscle.^73,74^ This multipotency makes BMSCs valuable for regenerative medicine applications, as they can be used to repair damaged tissues and organs. BMSCs can be isolated from bone marrow, and their differentiation can be directed toward specific cell lineages by providing appropriate growth factors and culture conditions.^74^ Thus, our analysis of BMSCs' functionality showed promoted differentiation into adipocytes and osteocytes (Fig. 5). As expected, both control and NF-free hAM groups exhibited baseline osteogenic differentiation under induction conditions, while NF-hAM treatment notably enhanced this effect. Furthermore, BMSCs underwent a morphological transition from fibroblastic to spherical during osteogenic induction, suggesting that NF-hAM preferentially directs BMSCs towards the osteogenic lineage rather than the adipogenic lineage. Moreover, the osteogenic inductive capacity of NF-hAM is greater than that of NF-free hAM and Ctrl under the given conditions. This coincides with the previous reports on MSCs cultured on D-hAM showing enhanced multilineage differentiation potential, including adipogenic and osteogenic differentiation.^75,76^

Elevated glucose flux through glycolysis supports cellular proliferation by providing additional ATP energy and metabolic intermediates for nucleotide, lipid, and protein biosynthesis.^77,78^ MSCs exhibit a metabolic phenotype during self-renewal characterized by increased glycolysis and decreased oxidative phosphorylation.^79^ This glycolytic metabolic profile is essential for maintaining their undifferentiated state and preserving their multipotent capacity.^80^ Glycolysis provides a rapid and efficient source of ATP, which is crucial for supporting the anabolic processes involved in cell proliferation and self-renewal.^80,81^ Additionally, glycolysis-derived metabolites can contribute to epigenetic modifications that help maintain a permissive chromatin environment for stem cell function.^82,83^ Hence, we studied glycolytic metabolism by assessing the glucose uptake of AMSCs, BMSCs, and HSFs from the conditioned medium and their capacity to produce lactate molecules in the conditioned medium. Our analysis shows that the glycolysis rate increases, as indicated by significantly decreased conditioned medium glucose levels and increased lactate production, which provides compelling evidence of enhanced glycolytic activity in the NF-hAM-treated cells.

In the NF-hAM group, β-actin staining is predominantly cytoplasmic and filamentous, suggesting a well-developed and organized actin cytoskeleton, while α-SMA staining is minimal, indicating a lack of differentiation towards a contractile phenotype. In contrast, the Ctrl group shows α-SMA positivity, particularly in a filamentous pattern, indicative of stress fiber formation. Concurrently, there seems to be a reorganization of β-actin with a more prominent stress fiber-like appearance (Fig. 5C). The observed decrease in α-SMA, a marker for contractile stress fibers, suggests potential modulation of the cytoskeleton that could affect cellular contractility (Fig. 5C).^84^ Interestingly, this decrease in α-SMA coincides with the enhanced metabolic activity observed in the MTT assay (Fig. 4). This could be indicative of a shift towards a more proliferative phenotype in NF-hAM-treated cells.^85^ Proliferating cells generally exhibit lower levels of α-SMA as they prioritize growth and division over contractility.^84,86^

PCNA (proliferating cell nuclear antigen) is a nuclear protein essential for DNA replication and repair. It acts as a sliding clamp for DNA polymerase *δ* during the S phase of the cell cycle. Upregulation of PCNA is a reliable indicator of active DNA synthesis and cellular proliferation. Its increase is crucial for expanding the cell population during tissue regeneration.^87^ Ki-67 is a nuclear protein present during all active phases of the cell cycle (G1, S, G2, and M), but absent in quiescent (G0) cells. It serves as a global marker of cell proliferation. Elevated Ki-67 expression confirms that the cells are not only cycling but are actively progressing through the cell cycle, reflecting an overall enhancement in growth kinetics.^88^ ID1 (inhibitor of differentiation 1) and ID2 are helix–loop–helix (HLH) proteins that function as dominant-negative regulators of lineage-specific bHLH transcription factors.^89^ By preventing these factors from binding to DNA, ID proteins inhibit premature differentiation and help maintain cells in an undifferentiated, proliferative state. ID1 has been shown to sustain the self-renewal of mesenchymal and embryonic stem cells and is often upregulated in rapidly proliferating progenitor cells. ID2 is particularly involved in neural and hematopoietic lineage regulation and contributes to maintaining a less differentiated state in stem and progenitor cells.^90^

Snail is a transcription factor that promotes EMT by repressing epithelial markers (like E-cadherin) and upregulating mesenchymal genes.^91^ It plays a central role in enabling cell migration and invasion, which are key processes in wound healing and tissue remodeling. Vimentin is a cytoskeletal intermediate filament protein that provides structural integrity and flexibility to migrating cells. It is a canonical marker of the mesenchymal phenotype and is strongly associated with increased motility and invasive capacity.^92^ VEGFA is a master regulator of angiogenesis, promoting endothelial cell proliferation, migration, and new blood vessel formation. Stem cells with high VEGFA expression are more effective in supporting vascular regeneration, which is critical for supplying oxygen and nutrients to healing tissues.^93^ PDGF stimulates the recruitment and proliferation of perivascular cells (*e.g.*, smooth muscle cells and pericytes) and contributes to vascular maturation and stabilization. It also has mitogenic effects on mesenchymal cells and enhances tissue regeneration.^94^

While our study demonstrates the promising biological effects of NF-hAM on MSCs and HSFs, the precise cellular uptake mechanisms of these nanoparticles remain to be elucidated. Future investigations should focus on identifying the pathways involved, such as clathrin-mediated endocytosis, caveolae-mediated endocytosis, and micropinocytosis as common routes for nanoparticle internalization. Employing pharmacological inhibitors, fluorescent labeling, and live-cell imaging could provide valuable insights into the cellular interactions and optimize the delivery efficiency of NF-hAM.

## 5. Conclusions

In conclusion, our study successfully developed novel NF-hAM NPs, harnessing the therapeutic potential of hAM proteins. The nanoparticles were characterized by their ultrastructure, thermal stability, and successful encapsulation of hAM proteins. Notably, NF-hAM demonstrated enhanced biocompatibility and efficacy in promoting stem cell proliferation, differentiation, and metabolic reprogramming. Our findings highlight the significant potential of NF-hAM as a promising nanomedicine approach for regenerative medicine. The targeted delivery of hAM proteins to stem cells through these nanoparticles offers a promising strategy for enhancing tissue repair and regeneration. Further research is warranted to explore the beneficial effect of NF-hAM nanoparticles on stem cell functions in enhancing wound healing and to evaluate their potential applications *in vivo*.

## Ethical statement and consent to participate

This study was conducted according to the guidance of the Institutional Review Board (IRB) ZU-IRB #11403 and the hAM samples were collected from patients after informed consent at Sheikh Zayed Hospital, Giza, Egypt.

## Consent for publication

All co-authors have given their consent to the submitted version of the manuscript for publication.

## Author contributions

AMA and MF conceptualized the study, design, and analysis of the results. AMA, MF, SS, DA, OI, and AK: development of methodology and acquisition of data. AMA, MF, DA, SS, and RAS: writing the first draft of the manuscript. NE has contributed to conceptualization, manuscript revision, and overall project administration. All authors read and approved the final manuscript.

## Conflicts of interest

The authors declare that there are no competing interests to disclose.

## Supplementary Material

NA-OLF-D5NA00222B-s001

## Data Availability

The data supporting this article have been included as part of the ESI.†
