# Supplementary material for: Amniotic membrane-encapsulated chitosan–lecithin nanoparticles promote the regenerative potential of mesenchymal stromal cells and fibroblasts
Source: Nanoscale Adv. 2025 Jun 26;7(16):5042–57. doi: 10.1039/d5na00222b (PMC12243162; doi:10.1039/d5na00222b)
Supplement: NA-007-D5NA00222B-s001 [file NA-007-D5NA00222B-s001.pdf]

Supplementary Information (SI) for Nanoscale Advances.
This journal is © The Royal Society of Chemistry 2025

# Supplementary file. 1

To investigate the cytoprotecting potential of NF-hAM from induced oxidative stress, HSF cells were exposed to H2O2 treatment at a final concentration of 300 μM for 12 hours, followed by 48 hours of NF-hAM treatment. Then, we studied their survival by MTT assay (Fig. 1) and resistance to apoptosis induction (Fig. 1). Our analysis demonstrated a significantly higher proliferation rate in the NF-hAM group as compared to $H_2O_2$-treated cells (Fig. 1).

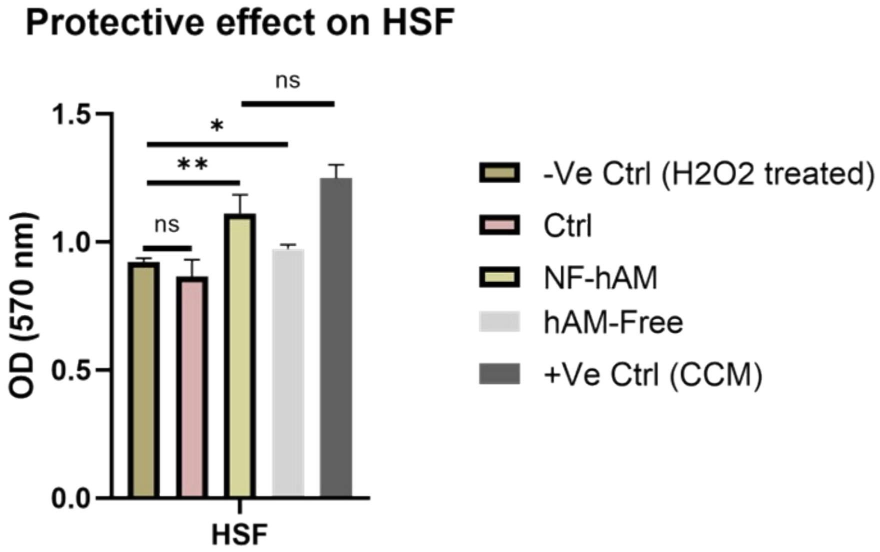


Fig. 1 NF-hAM treatment protects HSF from H2O2 cytotoxicity. MTT assay to investigate HSF proliferative capacity upon $H_2O_2$ treatment, followed by NF-hAM treatments.

Table 1: Primer sequence used in RT-qPCR analysis.

| Gene (protein) | Primer | Sequence (5' to 3') |
|---|---|---|
| β-actin | Forward | CACCATTGGCAATGAGCGGTTC |
| | Reverse | AGGTCTTTGCGGATGTCCACGT |
| ID 1 | Forward | GCTGTTACTCACGCCTCAA |
| | Reverse | CAACTGAAGGTCCCTGATGTAG |
| ID2 | Forward | CAAGAAGGTGAGCAAGATGGA |
| | Reverse | GGTGATGCAGGCTGACAATA |
| VEGFA | Forward | ATCTGCATGGTGATGTTGGA |
| | Reverse | GGGCAGAATCATCACGAAGT |
| PDGF | Forward | GATACTTTGCGCGCACACAC |
| | Reverse | GGTTTTCTCTTTGCAGCGAGG |
| Ki-67 | Forward | ACGAGACGCCTGGTTACTATC |

| | | |
|---|---|---|
| | Reverse | GCTCATCAATAACAGACCCATTTAC |
| PCNA | Forward | GGCCGAAGATAACGCGGATAC |
| | Reverse | GGCATATACGTGCAAATTCACCA |
| Snail | Forward | ACCACTATGCCGCGCTCTT |
| | Reverse | GGTCGTAGGGCTGCTGGAA |
| Vimentin | Forward | TGTCCAAATCGATGTGGATGTTTC |
| | Reverse | TTGTACCATTCTTCTGCCTCCTG |

## Raw data

| Table format: Grouped | | Group A | | | Group B | | | Group C | | | Group D | | |
|---|---|---|---|---|---|---|---|---|---|---|---|---|---|
| | | Ctrl | | | NF-hAM | | | NF-free hAM | | | hAM | | |
| | | A:1 | A:2 | A:3 | B:1 | B:2 | B:3 | C:1 | C:2 | C:3 | D:1 | D:2 | D:3 |
| 1 | BMSCs | 0.591 | 0.581 | 0.585 | 0.615 | 0.606 | 0.610 | 0.585 | 0.575 | 0.580 | 0.59 | 0.60 | 0.59 |
| 2 | HSF | 0.456 | 0.496 | 0.476 | 0.504 | 0.512 | 0.508 | 0.450 | 0.470 | 0.464 | 0.48 | 0.49 | 0.48 |
| 3 | AMSCs | 0.540 | 0.586 | 0.594 | 0.764 | 0.775 | 0.700 | 0.610 | 0.661 | 0.670 | 0.69 | 0.68 | 0.69 |
| 4 | Title | | | | | | | | | | | | |
| 5 | Title | | | | | | | | | | | | |

**Fig.2 A. raw data for** ***MTT analysis of BMSCs, AMSCs, and HSF under different experimental conditions***

| Table format: Grouped | | Group A | | | Group B | | | Group C | | |
|---|---|---|---|---|---|---|---|---|---|---|
| | | Ctrl | | | NF-hAM | | | NF-free hAM | | |
| | | A:1 | A:2 | A:3 | B:1 | B:2 | B:3 | C:1 | C:2 | C:3 |
| 1 | G0-G1 | 59.800 | 61.200 | 63.300 | 61.500 | 62.500 | 62.900 | 57.400 | 56.800 | 57.600 |
| 2 | S | 6.680 | 6.370 | 6.180 | 13.500 | 12.200 | 13.100 | 10.400 | 10.900 | 8.430 |
| 3 | G2-M | 13.100 | 14.400 | 15.500 | 13.200 | 12.400 | 12.500 | 13.500 | 10.900 | 10.400 |

**Fig. 2C raw data for** ***Bar graphs indicating BMSC population percentages in different cell cycle phases***

| Table format: Grouped | | Group A | | | Group B | | | Group C | | |
|---|---|---|---|---|---|---|---|---|---|---|
| | | Ctrl | | | NF-hAM | | | NF-free hAM | | |
| | | A:1 | A:2 | A:3 | B:1 | B:2 | B:3 | C:1 | C:2 | C:3 |
| 1 | Annexin V + PI - | 1.370 | 1.570 | 1.510 | 0.310 | 0.350 | 0.330 | 0.370 | 0.550 | 0.370 |

| Table format: Grouped | Group A Ctrl | | | Group B NF-hAM | | | Group C NF-free hAM | | |
|---|---|---|---|---|---|---|---|---|---|
| | A:1 | A:2 | A:3 | B:1 | B:2 | B:3 | C:1 | C:2 | C:3 |
| 1 Annexin V + PI + | 3.300 | 3.370 | 3.100 | 1.560 | 1.720 | 1.570 | 1.860 | 1.740 | 1.880 |

| Table format: Grouped | Group A Ctrl | | | Group B NF-hAM | | | Group C NF-free hAM | | |
|---|---|---|---|---|---|---|---|---|---|
| | A:1 | A:2 | A:3 | B:1 | B:2 | B:3 | C:1 | C:2 | C:3 |
| 1 Annexin V - PI + | 17.100 | 17.100 | 17.400 | 15.100 | 14.500 | 15.200 | 11.300 | 10.800 | 10.800 |

**Fig.3B, C, and D: apoptosis analysis**

| Table format: XY | X BMSCs | Group A NF-hAM | | | Group B NF-free hAM | | |
|---|---|---|---|---|---|---|---|
| | X | A:Y1 | A:Y2 | A:Y3 | B:Y1 | B:Y2 | B:Y3 |
| 1 Ctrl | | 0.138 | 0.135 | 0.150 | 0.138 | 0.135 | 0.136 |
| 2 5 μg/ml | | 0.167 | 0.165 | 0.166 | 0.156 | 0.151 | 0.154 |
| 3 50 μg/ml | | 0.173 | 0.166 | 0.165 | 0.160 | 0.148 | 0.155 |

**Fig. 4B** ***MTT assay for BMSCs treated with NF-hAM for 24 hours***

| le format: XY | X BMSCs | Group A NF-hAM | | | Group B NF-free hAM | | |
|---|---|---|---|---|---|---|---|
| | X | A:Y1 | A:Y2 | A:Y3 | B:Y1 | B:Y2 | B:Y3 |
| Ctrl | | 0.366 | 0.365 | 0.369 | 0.366 | 0.365 | 0.369 |
| 5 μg/ml | | 0.405 | 0.406 | 0.405 | 0.376 | 0.365 | 0.366 |
| 50 μg/ml | | 0.408 | 0.409 | 0.410 | 0.359 | 0.357 | 0.358 |

**Fig. 4D** ***MTT assay for BMSCs treated with NF-hAM for 48 hours***

| Table format: XY | X BMSCs | Group A NF-hAM | | | Group B NF-free hAM | | |
|---|---|---|---|---|---|---|---|
| | X | A:Y1 | A:Y2 | A:Y3 | B:Y1 | B:Y2 | B:Y3 |
| 1 Ctrl | | 0.388 | 0.385 | 0.382 | 0.388 | 0.385 | 0.382 |
| 2 5 μg/ml | | 0.412 | 0.416 | 0.414 | 0.384 | 0.388 | 0.385 |
| 3 50 μg/ml | | 0.423 | 0.426 | 0.425 | 0.383 | 0.397 | 0.390 |

**Fig. 4F** ***MTT assay for BMSCs treated with NF-hAM for 72 hours***

| Table format: XY | X HSF | Group A 24 hours | | | Group B 48 hours | | |
|---|---|---|---|---|---|---|---|
| | X | A:Y1 | A:Y2 | A:Y3 | B:Y1 | B:Y2 | B:Y3 |
| 1 Ctrl | | 0.263 | 0.257 | 0.251 | 0.376 | 0.369 | 0.373 |
| 2 5 μg/ml | | 0.264 | 0.271 | 0.268 | 0.401 | 0.416 | 0.405 |
| 3 10 μg/ml | | 0.271 | 0.272 | 0.272 | 0.416 | 0.400 | 0.410 |
| 4 50 μg/ml | | 0.275 | 0.277 | 0.280 | 0.436 | 0.415 | 0.425 |
| 5 Title | | | | | | | |

**Fig. 4I** ***MTT assay for HSF treated with NF-hAM for 24 and 48 hours***

| Table format: Grouped | | Group A | | | Group B | | | Group C | | |
|---|---|---|---|---|---|---|---|---|---|---|
| | | Ctrl | | | NF-hAM | | | NF-free hAM | | |
| | | A:1 | A:2 | A:3 | B:1 | B:2 | B:3 | C:1 | C:2 | C:3 |
| 1 | Glucose concentration | 0.682 | 0.679 | 0.655 | 0.142 | 0.139 | 0.148 | 0.258 | 0.256 | 0.295 |
| 2 | Title | | | | | | | | | |

**Fig. 6A** ***BMSCs glucose consumption***

| Table format: Grouped | | Group A | | | Group B | | | Group C | | |
|---|---|---|---|---|---|---|---|---|---|---|
| | | Ctrl | | | NF-hAM | | | NF-free hAM | | |
| | | A:1 | A:2 | A:3 | B:1 | B:2 | B:3 | C:1 | C:2 | C:3 |
| 1 | Lactate production | 0.501 | 0.504 | 0.505 | 0.556 | 0.534 | 0.547 | 0.510 | 0.510 | 0.508 |

**Fig. 6B** ***BMSCs lactate production***

| Table format: Grouped | | Group A | | | Group B | | | Group C | | |
|---|---|---|---|---|---|---|---|---|---|---|
| | | Ctrl | | | NF-hAM | | | hAM-Free | | |
| | | A:1 | A:2 | A:3 | B:1 | B:2 | B:3 | C:1 | C:2 | C:3 |
| 1 | H2O2 production | 0.123 | 0.125 | 0.131 | 0.084 | 0.082 | 0.077 | 0.076 | 0.082 | 0.078 |

***Fig. 6C HSF H2O2 production***

| Table format: Grouped | | Group A | | | Group B | | | Group C | | |
|---|---|---|---|---|---|---|---|---|---|---|
| | | Ctrl | | | NF-hAM | | | hAM-Free | | |
| | | A:1 | A:2 | A:3 | B:1 | B:2 | B:3 | C:1 | C:2 | C:3 |
| 1 | Glucose concentration | 1.443 | 1.462 | 1.363 | 1.117 | 0.955 | 1.181 | 1.308 | 1.083 | 1.206 |

***Fig. 6D HSF glucose consumption***

| Table format: Grouped | | Group A | | | Group B | | | Group C | | |
|---|---|---|---|---|---|---|---|---|---|---|
| | | Ctrl | | | NF-hAM | | | hAM-Free | | |
| | | A:1 | A:2 | A:3 | B:1 | B:2 | B:3 | C:1 | C:2 | C:3 |
| | Lactate production | 0.450 | 0.470 | 0.422 | 0.460 | 0.476 | 0.500 | 0.425 | 0.422 | 0.384 |

***Fig. 6E HSF lactate production***

| Table format: Grouped | | Group A | | | Group B | | | Group C | | |
|---|---|---|---|---|---|---|---|---|---|---|
| | | Ctrl | | | NF-hAM | | | hAM-Free | | |
| | | A:1 | A:2 | A:3 | B:1 | B:2 | B:3 | C:1 | C:2 | C:3 |
| 1 | Glucose concentration | 2.295 | 2.249 | 2.413 | 1.847 | 1.944 | 2.049 | 2.444 | 2.192 | 2.270 |

***Fig. 6F AMSCs glucose consumption***

| Table format: Grouped | | Group A | | | Group B | | | Group C | | |
|---|---|---|---|---|---|---|---|---|---|---|
| | | Ctrl | | | NF-hAM | | | hAM-Free | | |
| | | A:1 | A:2 | A:3 | B:1 | B:2 | B:3 | C:1 | C:2 | C:3 |
| 1 | Lactate production | 1.286 | 1.412 | 1.446 | 2.056 | 2.060 | 2.182 | 1.499 | 1.152 | 1.236 |

***Fig. 6G AMSCs lactate production***
